# Fostering EFL/ESL Students’ Language Achievement: The Role of Teachers’ Enthusiasm and Classroom Enjoyment

**DOI:** 10.3389/fpsyg.2021.781118

**Published:** 2021-11-01

**Authors:** Yang Liu, Mingjie Zhang, Xuetao Zhao, Fang Jia

**Affiliations:** ^1^College of Foreign Languages, Hebei Agricultural University, Baoding, China; ^2^Department of English, Hebei University of Chinese Medicine, Shijiazhuang, China

**Keywords:** positive psychology, classroom enjoyment, EFL/ESL students’ language achievement, teachers’ enthusiasm, constructive sensation

## Abstract

Thanks to the inflow of positive psychology (PP) in language education in general and language learning in particular, extensive consideration has been drawn to the role of emotion in second language acquisition (SLA). Enjoyment as a mutual constructive sensation experienced by students has engrossed academic attention. Likewise, teachers are redirected as the most remarkable figure of any educational association, and their enthusiasm is substantial for students in the classroom. In line with the inquiries of teacher enthusiasm, principles of PP, and classroom enjoyment, the current review strives for this form of connection and its impacts on learners’ achievement. Subsequently, the suggestions of this review for teachers, learners, and educator trainers are deliberated.

## Introduction

A central objective of colleges is learners’ achievement and success, whose administration routes should pay great attention to observing learners’ educational performance. As a result, through institutional- and framework-level administration provisions, the quality of learners’ education should be taken into consideration (Jones, 2013). Generally measured with tests, scholastic achievement alludes to what exactly is done under existing conditions that incorporates the most common way of editing and using the construction of information and capacities and a large group of emotional, inspirational, and complex factors that impact definitive reactions (O’Donnell and White, 2005). Academic achievement is defined as the perceived and assessed part of a learner’s mastery of abilities and subject materials as estimated with legitimate and valid tests (Joe et al., 2014). Based on Nurhasanah and Sobandi (2016), there might be two kinds of factors, internal and external, which influence learning performance and learning success. In addition to issues, such as health, impairments, and cognitive elements (intelligence, aptitude, enthusiasm, concentration, motivation, and fatigue). While learners’ academic achievement and success are influenced by many external elements, including family members, educational environments, and cultural considerations, learner performance and achievement will be affected by these two internal and external elements.

On the significance of feelings in language learning, a great amount of the literature mentioned that feelings assume an indispensable part in students’ performances in a foreign language (MacIntyre and Vincze, 2017; Shao et al., 2019). There has been developing attention to feelings in language settings, all the more explicitly, in the way they influence language students’ motivation, praise, interest, commitment, practice, accomplishment, and well-being (Dewaele et al., 2019; MacIntyre et al., 2019; Dewaele and Li, 2021). Additionally, charged by the introduction of positive psychology (PP) in second language acquisition (SLA; MacIntyre and Gregersen, 2012) was the term “affective turn” (Pavlenko, 2013; Prior, 2019). Past research acknowledges the effect of negative emotions on language accomplishment (Ostafin et al., 2014; Ford et al., 2019).

Nonetheless, dependent on the PP development in instruction, there has lately been a growing extent of the literature on constructive emotions in SLA (Dewaele and MacIntyre, 2014; MacIntyre et al., 2019; Wang et al., 2021) provoking analysts to move their enduring attention from negative emotions (L2 stress and boredom) to positive ones (L2 satisfaction; Khajavy et al., 2018; Dewaele et al., 2019; Derakhshan et al., 2021; Dewaele and Li, 2021). As opposed to negative emotions, which trigger limited attitudes, positive emotions encourage the expansion of mentalities and discovery of inventive and novel notions, which can prompt the foundation of one’s physical, mental, scholarly, and social assets (Fredrickson, 2004). Moreover, positive emotions are helpful for individual investigation, permitting one to procure new experiences and learn successfully (Dewaele and MacIntyre, 2014; Xie and Derakhshan, 2021).

Accordingly, the range of emotion has been extended past stress to incorporate happiness, love, pride, trust, guilt, disgrace, fatigue, outrage, disappointment, and so on (Kruk and Zawodniak, 2018; Pavelescu and Petric, 2018; MacIntyre et al., 2019). Among all types of emotions, either negative or positive inspected in the language research trend, Foreign Language Enjoyment (FLE) has been viewed as the most regularly knowledgeable emotional aspect for students (Piniel and Albert, 2018). Classroom enjoyment is perceived as the degree to which L2 learning is regarded as providing joy (Dewaele and MacIntyre, 2014), which is a constructive emotional state that consolidates challenge, bliss, interest, fun, feeling of pride, and feeling of importance. It happens particularly in exercises where students have a level of independence and when something new or difficult is accomplished (Csikszentmihalyi and Seligman, 2000). FLE had jointly caught great attention for its critical ramifications for L2 results (Jin and Zhang, 2018; Li et al., 2018). It was figuratively theorized as the emotional feet of each L2 student for their prevalence in the L2 learning setting (Dewaele and MacIntyre, 2016). In a foreign language setting, enjoyment was picked as a positive equivalent of the broadly studied negative emotion of FLA generally since it is a center part of the foundational notion of PP, i.e., flow (Csikszentmihalyi, 2014).

Furthermore, supported by the broaden-and-build hypothesis, enjoyment is a progressive and emotion-focused action, one that decidedly impacts students’ scholarly presentation and it is an idea that resounds with the arising field of PP (Pekrun et al., 2007; Pekrun and Perry, 2014). The primary principle of broaden-and-build is that positive emotion, like enjoyment, can expand people’s thought-action collections and create their mental versatility and individual assets (Fredrickson, 2003; Oxford, 2015). Regarding SLA, it is contended that students encountering constructive emotions will assimilate more information and will create more assets for more language education. Conversely, negative emotions will limit students’ concentration and the scope of possible language input. Enjoyment, however, is effective in expanding students’ thought-action collection to assimilate more in language learning and assist them with building language assets (MacIntyre and Gregersen, 2012). In addition, according to the control-value hypothesis (Pekrun, 2006), FLE is a constructive accomplishment emotion with high motivation emerging from progressive learning action or assignment. It has constructive outcomes for different L2 learning results encompassing L2 motivation, commitment, and learning accomplishment (Li, 2020; Dewaele and Li, 2021). Since FLE urges students to be innovative and investigate a new language, it triggers foreign language learning (Dewaele and MacIntyre, 2016). Numerous past studies have additionally discovered that enjoyment is commonly connected with less stress and higher educational fulfillment (Dewaele and Dewaele, 2017). Latest movements in PP, nonetheless, have prompted an expansion of studies intended to stimulate the significance of language erudition being triggered by positive emotions, regarding the latter as an enhancer and the main impetus behind SLA (Oxford, 2015). It has been discovered up to date that FLE is positively connected with high scholarly achievement and proficiency in a foreign language (Hagenauer and Hascher, 2014; Dewaele and Alfawzan, 2018; Dewaele et al., 2019).

Moreover, in comparison with classroom enjoyment, teacher enthusiasm had a noteworthy impact on student-perceived teaching quality. The attitude toward something, generally a nonverbal practice of expressiveness, is known as enthusiasm. Thus, it is characterized as learners’ positive affective qualities, satisfaction, and joy during learning (Kunter et al., 2011). It is a quality-like, constant, and repeating feeling. Since enthusiasm pushes the learner to study and to receive the new cycle being experienced, it cannot be separated from a learning cycle. With enthusiasm, the learners show their delight in learning English by displaying their joyful facial expressions in the class and have a positive sentiment to learning a language. When learning something new like the English language, learners get excited. Enthusiasm can positively impact learners’ results (Patrick et al., 2000). The greater the learners’ enthusiasm in learning a language, the greater the results and the achievements they will encounter in learning.

Enthusiasm, as a significant quality for everyone, pays little heed to the type of work being done. To put it simply, an enthusiastic individual is a person who, in a real sense, is motivated by a strong force. Moreover, the motivated individual comes to perceive himself as a distinguished top pick of the divine nature. When this craze happens, which is the culmination of energy, each fanciful notion is accentuated (Nur, 2019). Educator enthusiasm could be characterized as the occurrence of assorted behavioral articulations, like, nonverbal (gestures) and verbal (tone of voice) practices (Keller et al., 2016).

The prior inquiries were conducted with respect to FLE in higher education (Dewaele et al., 2016; Dewaele and MacIntyre, 2016; Elahi Shirvan and Talebzadeh, 2018; Li et al., 2018; Talebzadeh et al., 2020). In addition, former studies have presented that teacher enthusiasm is connected to diverse constructive consequences, such as students’ enjoyment, interest, achievement, motivation, and vitality (Patrick et al., 2000; Kunter et al., 2013; Keller et al., 2014; König and Jucks, 2019). Although these investigations have been done in these domains, this review makes an effort to inspect the function of classroom enjoyment and teacher enthusiasm on learners’ achievement in foreign language learning.

### Teacher Enthusiasm

Perceived to be a fundamental component for enhancing teaching results is educator enthusiasm, which is quite possibly the main attribute of successful teachers (Kunter et al., 2011; Ruzek et al., 2016). Enthusiasm is a feeling based on sensory perception, providing the ability to detect, acknowledge, evaluate, or respond physically to something. A powerful stimulus can create excitement, which results in enjoyment or satisfaction in a particular activity. These definitions are described in terms of their causes and effects (Setianingsih and Nafisah, 2021). There is a theoretical explanation that enthusiasm promotes the attention of educators to their learners in the classroom (Kunter and Holzberger, 2014) and enhances constructive effect in learners by sharing emotive responses (Frenzel et al., 2018) resulting in improved learner relationships and a reduction in conflict (Kunter et al., 2013). Teacher enthusiasm is delineated as a nonverbal manifestation that the educator echoes (Baloch and Akram, 2018); teacher performances like presenting effective demonstrations, facilitating interaction, and communication (Hadie et al., 2019; Wang and Derakhshan, 2021); teachers’ distinctive features in line with the constructive emotional state (Keller et al., 2018); and constructive emotive practices that the educator achieves while carrying out her/his responsibility (Keller et al., 2014).

Enthusiasm is an individual’s feeling of getting energized, while acting and it is developed when she/he begins to be interested in the activity. Moreover, it can be characterized as a strong liking for a subject of matter, something, or activity, and a searing soul, fuel, or the blasting fire of something new. Learners regard enthusiasm as the focus of their learning and a prerequisite for their involvement (Pithers and Holland, 2006). Moreover, Kunter et al. (2011) categorized enthusiasm as an emotional-behavioral educator quality. The multiple-dimensional structure includes both a learner’s sense of pleasure, enjoyment, and interest, as well as specific learning activities that promote these feelings in the educational setting. Moreover, it was found that teachers’ enthusiasm for the subject was distinct from their enthusiasm for teaching activities. Keller et al. (2016) described educator engagement as both an emotional and cognitive quality consisting of a favorable psychological state, a sense of enjoyment, and happiness, as well as the exhibiting of specific behaviors (primarily physical) derived from them. Feeling enthusiasm and showing enthusiasm constitute enthusiasm, respectively. By proposing that educator enthusiasm is the emotional experience of pleasure during learning as well as its actions or expression during education, Frenzel et al. (2009) developed this theory. Educators’ expressions of enjoyment include smiles, widening eyes, and a mutable tone, and greater speed. Teacher enthusiasm that could be transferred to learners, boost their enthusiasm, participation, and commitment (Lazarides et al., 2019).

### Enjoyment

Enjoyment occurs when learners perceive themselves to be competent at performing an educational task as well as recognizing the content of the learning process (Mierzwa, 2019). An enjoyment construct comprises five categories: behavioral, mental, emotional, expressional, and psychological (Hagenauer and Hascher, 2014). In the same way that the name implies, the affective component of enjoyment focuses on emotions, and in particular, on the sense of satisfaction and pleasure felt during the learning process. Additionally, the mental aspect of learning is concerned with evaluating the situation positively. Hence, enjoyment could be considered the feeling of accomplishment that arises when completing a challenging, complex task that encourages inquiry (Pekrun et al., 2007) and creates enthusiasm (Ainley and Hidi, 2014; Han and Wang, 2021). Moreover, the motivational aspect of enjoyment refers to learners’ ability to feel good by encouraging them, emotionally and physically, to attempt more FL tasks in the future (Villavicencio and Bernardo, 2013). Since Broaden-and-Build Theory of constructive emotions of Fredrickson (2001) and the Control-Value Theory of emotions (Pekrun and Perry, 2014) both explain how positive emotions appear, it is logical to expect that FLE will perform similarly within the FL context and is deemed as a major source of positive achievement feelings that are directly linked with the enjoyment of flow.

The concept of FLE was introduced by MacIntyre and Gregersen (2012), explaining how the construct of enjoyment as a feeling associated with achievement could help learners develop resources to learn English more effectively (Pekrun, 2006), enhance their understanding, and improve their motivation in academic learning (Jin and Zhang, 2019). FLE is significantly affected by its educational process, such as the connections with thoughtful and cooperative classmates, as well as the communication with enthusiastic language educators that provide a variety of engaging and challenging classroom activities to engage learners (Pavelescu and Petric, 2018). There are two scopes of enjoyment in FLE: first, FLE is linked to the educator (teaching methods, encouragement, optimism, and educator acknowledgment); second, FLE is associated with the environment in FL education (social contacts, enthusiasm, and motivation; Li et al., 2018). A third dimension that is not less important than the two aforementioned is FLE-private coalescing with its contribution to personal development and personal progress. Among the sources of FLE-private, the following can be eminent: realizing one’s progress, achieving great FL results, achieving success, and observing improvements in FL learning (Li et al., 2018).

## Implications and Future Directions

This review has provided some suggestions for language stakeholders. It can be of significance for educators, pupils, and educator trainers. Various studies have found that FLE is a motivational force that impacts language learning in several ways as it enables higher academic achievement, enhances motivation, and may even protect individuals against negatively framed views (MacIntyre, 2016). In this sense, FLE provides a useful experience for educational purposes and might be essential for learners to achieve full proficiency in multiple languages. Corresponding to the Control-Value Theory, learners’ ability to internalize values of academic engagement and achievement is likely to be enhanced when their educators demonstrate enthusiasm and enjoyment regarding a particular subject or learning activity (Pekrun, 2006; Derakhshan, 2021).

The present review can be valuable for language learners as they can have less anxiety and become more confident and autonomous when facing challenges in the classroom when their educators are enthusiastic. It has been found that enthusiasm can have a major impact on educational success, so it is evident that enthusiasm has a significant effect on learners’ performance and helps them to be successful in academic learning. The educator’s enthusiasm ensures they intend to go above and beyond to fulfill their professional duties, as well as give the correct clues to their learners. As a result, an enthusiastic educator can be defined as one who seeks to accomplish the teaching and learning process effectively and performs his or her duty of supporting the learners as needed for successful teaching and learning. Furthermore, it is the teacher’s concern to control the emotional atmosphere of the classroom, to nurture a positive sense among the students, and, ideally, to teach with excitement, enthusiasm, and interest (Dewaele et al., 2018).

The enthusiasm of the educators will make a difference not only on the learning side, but also during the educational process since educators organize these activities (Bedir and Yıldırım, 2000). An educator should be enthusiastic when performing the teaching duties given to her/him. An educator should adjust the course and tone of voice according to the learners’ interests and abilities during the presentation to ensure that the course is engaging for the learners and demonstrates their enthusiasm for the course (Keller et al., 2016).

Learners who are imitating an enthusiastic educator are likely to acquire the educator’s outlook concerning interest, motivation, and beliefs, leading to enhanced knowledge and a positive attitude toward education (Keller et al., 2016). Developing excitement and enjoyment in teaching and the subject area should be a central element of educators’ training. Additionally, an atmosphere that allows educators to maintain enthusiasm is necessary during their daily work. For example, educators can avoid stressful working conditions by reducing organizational workloads and management responsibilities. Finally, it may be possible to maximize learners’ interest, motivation, and achievement by enhancing educators’ enthusiasm. Throughout the course, letting the learners speak and share their perspectives, engaging, giving advice regarding the accuracy or errors of their statements, and addressing their mistakes are factors that contribute to the enthusiasm of the teacher that leads to students’ achievement. To include the perspectives of other professionals in the field of education, it would be useful to conduct further research on the topic of teacher enthusiasm. Studies involving educators with different levels of knowledge, rather than just new educators, could help to evaluate enthusiasm levels. Additionally, investigators can conduct studies on a national and international basis, regardless of their academic background.

## Author Contributions

All authors listed have made a substantial, direct and intellectual contribution to the work, and approved it for publication.

## Conflict of Interest

The authors declare that the research was conducted in the absence of any commercial or financial relationships that could be construed as a potential conflict of interest.

## Publisher’s Note

All claims expressed in this article are solely those of the authors and do not necessarily represent those of their affiliated organizations, or those of the publisher, the editors and the reviewers. Any product that may be evaluated in this article, or claim that may be made by its manufacturer, is not guaranteed or endorsed by the publisher.
